# Epstein–Barr Virus and Multiple Sclerosis: A Narrative Review on Prevention and the Concept of an Infection-Driven Disease

**DOI:** 10.3390/biomedicines14050962

**Published:** 2026-04-22

**Authors:** Lou Marie Salomé Schleicher, Dorotea Zivalj, Hadid Joseph Farzad Diamee, Jan Finderle, Antea Krsek, Lara Baticic

**Affiliations:** 1 Faculty of Medicine, University of Rijeka, 51000 Rijeka, Croatia; loums.schleicher@uniri.hr (L.M.S.S.); dzivalj@uniri.hr (D.Z.); hadid.diamee@uniri.hr (H.J.F.D.); jan.finderle@uniri.hr (J.F.); tea.krsek@gmail.com (A.K.); 2 Department of Medical Chemistry, Biochemistry and Clinical Chemistry, Faculty of Medicine, University of Rijeka, 51000 Rijeka, Croatia

**Keywords:** Epstein–Barr virus infections, multiple sclerosis, neuroinflammatory diseases, risk factors, vaccination

## Abstract

Multiple sclerosis (MS) is a chronic inflammatory disease of the central nervous system with onset typically in early adulthood and the potential for long-term disability. Current therapies are initiated after symptom onset and do not address early disease triggers, highlighting the need for preventive strategies. Epstein–Barr virus (EBV) infection has emerged as the strongest candidate upstream factor in MS development. This narrative review provides a focused and critical synthesis of current evidence, with particular emphasis on the prevention perspective and the conceptual framing of MS as a potentially infection-driven disease. We integrate epidemiological, immunological, and mechanistic data while explicitly addressing key uncertainties and limitations in causal interpretation. Longitudinal studies indicate that EBV infection precedes MS onset in most cases and is associated with a markedly increased risk following seroconversion. However, EBV infection alone is not sufficient to cause MS. Proposed mechanisms include immune dysregulation and molecular mimicry, though key uncertainties remain. Based on current evidence, EBV represents a promising but unproven target for MS prevention. Future strategies may include prevention of EBV infection or infectious mononucleosis, alongside improved risk stratification and long-term studies to assess the impact of EBV-targeted interventions on MS incidence.

## 1. Introduction

Multiple sclerosis (MS) is a chronic inflammatory disease of the central nervous system characterized by immune-mediated damage, neuroaxonal loss, and progressive disability [1,2]. It is widely considered a multifactorial disorder, arising from the interplay between genetic susceptibility, environmental exposures, and immune dysregulation. The traditional autoimmune model emphasizes aberrant immune responses against central nervous system components, supported by genetic associations such as HLA related risk loci. In parallel, environmental factors, including vitamin D deficiency, smoking, and viral infections, have been consistently implicated in disease risk. Within this framework, increasing attention has been given to Epstein–Barr virus (EBV) as a potential contributing factor in MS pathogenesis. Rather than representing a single-cause explanation, EBV is increasingly viewed as one component within a complex network of interacting influences that may trigger disease in susceptible individuals [1,2,3,4,5]. This perspective allows integration of infectious, immunological, and genetic models, rather than replacing established concepts of MS as a multifactorial disease. This narrative review aims to summarize and critically evaluate current epidemiological, immunological, and mechanistic evidence linking EBV to MS, while highlighting key limitations, knowledge gaps, and potential implications for prevention strategies.

### 1.1. Key Facts and Players in Multiple Sclerosis Initiation

#### 1.1.1. Clinical and Biological Framework of Multiple Sclerosis

MS is a chronic autoimmune disorder of the central nervous system (CNS), characterized by inflammation, demyelination, gliosis, and neuroaxonal loss [1]. It typically manifests in early adulthood, with a mean age at diagnosis of approximately 32 years, often affecting individuals during key phases of education, career development, and family formation [2]. Even in the early stages, symptoms such as fatigue and cognitive impairment may substantially impact daily functioning [3].

MS is clinically heterogeneous and classified into distinct phenotypes, including relapsing-remitting MS (RRMS), secondary progressive MS (SPMS), and primary progressive MS (PPMS), as well as early-stage entities such as clinically isolated syndrome (CIS) and radiologically isolated syndrome (RIS) [4,5]. RRMS represents the most common initial course, characterized by relapses followed by remission, whereas SPMS and PPMS reflect progressive accumulation of disability [6]. RIS and CIS are increasingly recognized as part of a preclinical or early disease continuum with measurable risk of progression [7,8].

**Figure 1:**
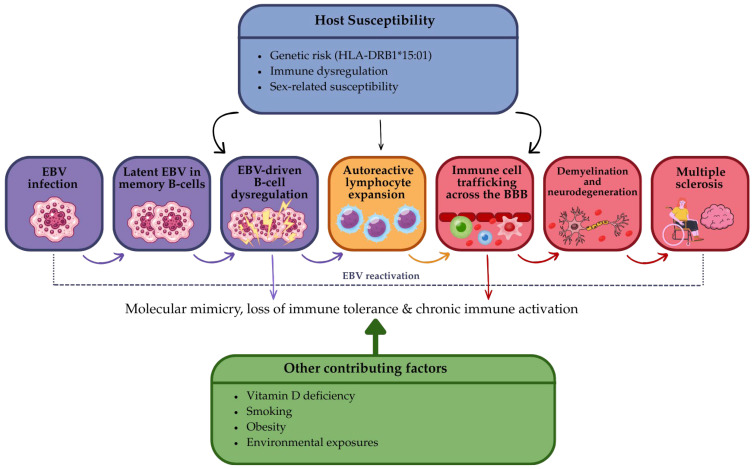
Schematic model of EBV-driven multifactorial mechanisms in multiple sclerosis pathogenesis. EBV establishes latency in memory B cells, promoting B-cell dysregulation and autoreactive lymphocyte expansion. In genetically susceptible individuals, and in the presence of environmental modifiers, these processes facilitate immune cell trafficking across the blood–brain barrier, leading to neuroinflammation, demyelination, and neurodegeneration characteristic of multiple sclerosis. Feedback mechanisms, including EBV reactivation, may further sustain immune dysregulation and disease progression. (BBB—blood–brain barrier; EBV—Epstein–Barr virus).

This phenotypic diversity reflects the multifactorial nature of MS, which arises from the interaction between genetic susceptibility, environmental exposures, and immune dysregulation. Within this framework, EBV has emerged as a potential upstream factor, but its role must be interpreted in the context of this broader disease model. Figure 1 illustrates the current concept of EBV-driven mechanisms within a multifactorial MS framework.

#### 1.1.2. Immune Mechanisms and Compartmentalized Inflammation

MS pathogenesis involves a complex interplay of adaptive and innate immune responses. T cells contribute to CNS infiltration and inflammatory amplification, while B cells act not only as antibody producers but also as antigen-presenting and cytokine-secreting cells [9]. Microglia and infiltrating macrophages further sustain local inflammation and contribute to neurodegeneration.

Importantly, MS is increasingly understood as a disease of compartmentalized inflammation, in which immune activity persists within CNS niches such as the meninges and perivascular spaces, even when peripheral inflammatory activity is suppressed [10]. This concept provides a key framework for understanding disease progression and therapeutic limitations.

#### 1.1.3. Limitations of Current Therapeutic Paradigms

Disease-modifying therapies (DMTs) have significantly improved MS outcomes, particularly in relapsing disease. However, these treatments are initiated after clinical onset and primarily target inflammatory activity rather than disease initiation [11]. A major limitation is the persistence of disability progression independent of relapse activity (PIRA), which is thought to be driven by compartmentalized CNS inflammation [12]. This highlights a critical unmet need: the identification of upstream disease drivers and the development of preventive strategies, rather than solely reactive treatment approaches.

## 2. Epstein–Barr Virus in the Context of MS Pathogenesis

### 2.1. Epstein–Barr Virus Biology: Relevance and Limitations

Epstein–Barr virus (EBV), a member of the Herpesviridae family, is nearly universally acquired, with adult seroprevalence exceeding 90% [13]. It primarily infects B lymphocytes and establishes lifelong persistence following primary infection [14]. EBV alternates between lytic and latent infection states. The lytic phase involves active replication and viral shedding, whereas latency is characterized by restricted viral gene expression and persistence within memory B cells [15]. While these biological features provide a plausible framework for long-term immune interaction, their direct relevance to MS remains largely inferential, as most supporting evidence is derived from observational or experimental models rather than direct human tissue studies.

### 2.2. EBV as a Candidate Upstream Factor in MS

Longitudinal studies demonstrate that EBV infection precedes MS onset in the vast majority of cases and is associated with a markedly increased risk following seroconversion [16]. These findings support EBV as a strong candidate trigger of disease. However, EBV infection alone is not sufficient to cause MS, and its role may vary between individuals. It may act as a trigger, modifier, or part of a broader set of interacting risk factors rather than a single causal agent. This distinction is critical when interpreting associations and designing prevention strategies.

### 2.3. Primary Infection Phenotype and Risk Modification

The clinical phenotype of primary EBV infection appears to influence MS risk. While infection in childhood is often asymptomatic, delayed infection may result in infectious mononucleosis (IM), which is associated with increased MS risk [17]. Mechanistically, IM reflects a more pronounced immune response, including strong EBV-specific T-cell activation and systemic inflammation [17,18,19,20,21]. Some studies suggest that this may leave a long-lasting immunological imprint [22]. However, direct evidence linking these immune changes to MS pathogenesis remains limited, and the observed associations may reflect broader host–environment interactions rather than a single causal pathway.

### 2.4. EBV Persistence, Immune Interaction, and Mechanistic Uncertainty

EBV persistence is maintained through its ability to exploit normal B-cell biology. After infecting naïve B cells, EBV can promote their entry into germinal center reactions and differentiation into memory B cells [23,24,25]. Although this process is well described in experimental systems, its role in MS remains conceptual rather than directly demonstrated. The presence of a long-lived EBV reservoir provides a plausible mechanism for chronic immune modulation, but whether it actively drives autoimmune processes or represents background viral persistence is unresolved. Similarly, EBV employs multiple immune evasion strategies, including reduced antigen expression and interference with antigen presentation [13,26]. While these mechanisms support viral persistence, their specific contribution to MS-related immune dysregulation is not clearly established and may overlap with broader immune abnormalities observed in the disease [27].

### 2.5. Reactivation, Measurement Challenges, and Conflicting Evidence

EBV reactivation is often proposed as a contributing mechanism, but evidence in humans remains inconsistent. Current approaches rely on indirect markers such as serology or detection of EBV DNA in blood [15,28]. These measures have important limitations. Antibody responses may persist long-term and fluctuate over time [29], while EBV DNA detection may reflect latent infection rather than active replication [30]. Furthermore, results vary depending on specimen type and methodology [31]. As a result, there is no reliable in vivo marker of EBV reactivation in disease-relevant compartments, and its role in MS remains uncertain.

### 2.6. Integrative Interpretation: What Remains Unresolved

A key unresolved question is whether EBV contributes to MS primarily through latency, reactivation, or broader immune modulation. Current evidence supports a model in which EBV is a plausible upstream contributor within a multifactorial disease framework, rather than a definitively established causal agent. Latency-based models are supported by biological plausibility but lack direct in vivo validation, while reactivation-based hypotheses remain inconsistent across studies. Intrathecal EBV-specific responses are generally weak, further complicating interpretation [32,33]. Overall, the EBV–MS relationship is best understood as associative and mechanistically plausible, but not yet causally proven, with significant gaps remaining in human evidence. Table 1 summarizes key EBV infection biology checkpoints relevant to neuroimmune disease.

## 3. Multiple Sclerosis Immunopathogenesis Through the Lens of Epstein–Barr Virus

### 3.1. B Cells as Antigen-Presenting and Cytokine-Producing Nodes

**Figure 2:**
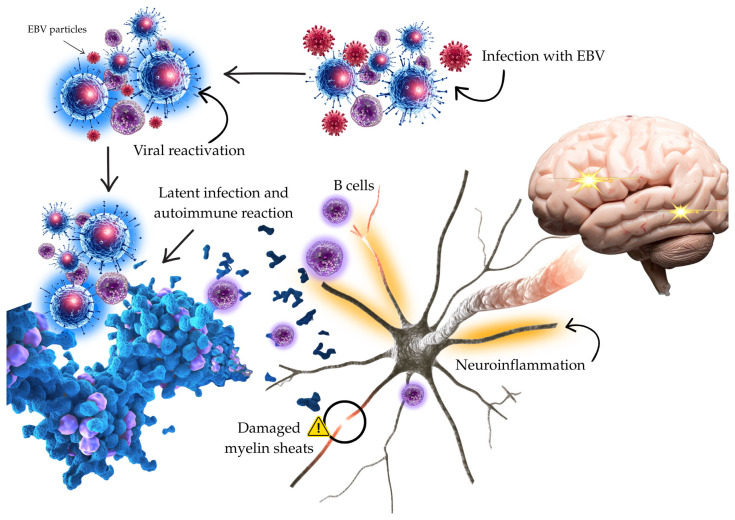
Conceptual schematic linking Epstein–Barr virus persistence in B cells to neuroinflammation and demyelination in multiple sclerosis. (EBV—Epstein–Barr virus).

In the past decade, research on the immunopathogenesis of MS has increasingly focused on B cells as active immune organizers rather than solely antibody producers. In both peripheral tissues and the CNS, B cells take up antigen through the B-cell receptor, process it, and display peptide fragments on MHC class II molecules, enabling engagement with CD4+ T-cells. Additionally, B cells secrete cytokines that influence the local immune environment, promoting either proinflammatory or regulatory responses. The presence of B-cell aggregates in some patients has been associated with inflammatory cortical pathology [32]. This framework helps explain why B-cell-depleting therapies are effective in relapsing MS. Anti-CD20 monoclonal antibodies substantially reduce relapse rates and MRI disease activity by depleting CD20+ B-cell subsets, including memory B cells, while largely sparing antibody-secreting plasma cells. This apparent disconnect supports the view that much of the therapeutic benefit comes from disrupting antibody-independent B-cell functions—particularly antigen presentation, cytokine-mediated signaling, and B–T-cell interactions—rather than simply lowering circulating antibody levels [33]. In this context, EBV is relevant not because anti-CD20 therapy proves causality, but because it highlights the B-cell compartment as a key immunological bottleneck. The fact that EBV resides long-term in B cells raises the possibility that infected B cells can modulate antigen presentation or cytokine signaling. However, the effectiveness of anti-CD20 therapy should be viewed as evidence of B-cell involvement in MS pathogenesis, not as direct proof that EBV is the initiating cause (Figure 2) [37]. The interpretation of the EBV and MS relationship should take into account differences in the strength of available evidence. Large longitudinal studies support a clear temporal association, while proposed mechanisms such as latency, molecular mimicry, or reactivation remain mostly based on indirect or experimental data and are not fully proven. In addition, alternative explanations such as general immune dysregulation, genetic factors, and environmental influences may also contribute to these findings. Therefore, EBV should be considered a strong contributing factor within a multifactorial model rather than a single causal explanation.

### 3.2. T-Cell Polarization, Central Nervous System Entry, and Compartmentalized Inflammation

In the pathobiology of MS, T cells remain central as effectors within lesions and additionally as early gatekeepers that shape peripheral activation and CNS-directed trafficking. Current research emphasizes that T-cell responses relevant to MS develop in the periphery, where encounters with antigens and inflammation program effector functions and capacity for trafficking. Within this framework, Th1- and Th17-skewed CD4+ T cells, cytotoxic CD8+ T cells, and inadequate Treg counter-regulation contribute to demyelination and neurodegeneration. Barrier interactions facilitate their entry into the CNS [40]. When activated T cells are adequately programmed, they can interact with the BBB and blood–CSF interfaces via coordinated adhesion and signaling events, thereby facilitating transmigration and amplifying local inflammation [41]. One well-known way this process is amplified is through GM-CSF (Granulocyte–Macrophage Colony-Stimulating Factor) production by pathogenic helper T cells. They enable promotion of myeloid activation and escalation of neuroinflammatory cascades [42]. Over time, MS inflammation may become partially compartmentalized within the CNS. This helps explain why disability can progress despite suppressed peripheral inflammation. Recent research in progressive biology indicates that immune activity persists within perivascular and leptomeningeal spaces. Chronic activation states and organized immune cell aggregates, including tertiary lymphoid structure-like formations in certain patients, are associated with more severe cortical pathology and disease progression. This compartmentalized configuration suggests that early immune programming and CNS entry may be more preventable than entrenched, self-sustaining inflammation once chronic niches are established [10]. Within an infection-driven framework, EBV is relevant to the T-cell compartment in a specific manner. The virus persists in B cells, which serve as key antigen-presenting and cytokine-producing partners for T cells. MS is associated with altered adaptive immune signatures against EBV, including EBV antigen-specific T-cell responses. These observations support a plausible pathway in which EBV-conditioned B-cell–T-cell interactions could influence peripheral programming and, downstream, CNS-directed immunity. At the same time, it remains unclear whether EBV acts primarily as a trigger, a modifier of immune set-points, or a contributor to persistence in a subset of patients [38].

### 3.3. Meningeal Inflammation and Progressive Biology

Progressive disability in MS is increasingly linked to CNS-compartmentalized inflammation that persists behind a largely repaired BBB, reducing the relevance of peripheral relapse activity as the main readout of ongoing pathology [10]. A defining hallmark of this compartmentalized biology is leptomeningeal inflammation, which ranges from diffuse cellular infiltrates to more organized immune aggregates often discussed as tertiary lymphoid structures (TLSs) or ectopic follicle-like formations [10]. From the perspective of disease progression, this meningeal compartment is frequently cited to explain progression independent of relapse activity (PIRA) and the limited impact of many relapse-focused endpoints on long-term disability [43]. In experimental models of leptomeningeal TLS-like inflammation, Th17-associated pathways, including IL-17 and GM-CSF, contribute to the development and maintenance of chronic meningeal immune niches linked to gray-matter injury [44]. Because direct sampling of meningeal pathology is difficult in living patients, in vivo work has leaned on imaging and fluid proxies, with important caveats [45]. Leptomeningeal enhancement (LME) observed on MRI is commonly regarded as an indicator of leptomeningeal inflammation. However, LME is not specific to MS, shows considerable variability across studies and MRI field strengths, and is more frequently reported in progressive than in relapsing forms of the disease [46]. LME also appears relatively stable over time in many datasets, and to date, relatively few therapies have shown consistent effects on LME burden. Therefore, it seems that at least part of the leptomeningeal process is pharmacologically harder to reach than peripheral inflammatory activity [46]. In parallel, CSF markers linked to follicle-like inflammation, most notably CXCL13-related biology, are used as enrichment or monitoring candidates, but their clinical interpretation remains context-dependent and imperfect [47]. These limitations are one reason why recent trial methods emphasize developing outcomes tailored to compartmentalized inflammation, such as meningeal inflammation and chronic active lesion metrics, rather than relying solely on new or enhancing white-matter lesions [45]. This framework matters for EBV because B-cell-rich meningeal compartments are mechanistically compatible with hypotheses in which EBV-influenced B-cell states contribute to chronic, CNS-restricted immune activity. Yet the neuropathologic evidence for EBV within the MS CNS and meningeal niches has been heterogeneous and method-dependent, and the field remains divided on how often and where EBV can be demonstrated in tissue [48]. Contemporary models of EBV in MS increasingly distinguish between early causal roles, such as triggers or necessary factors, and potential late driver roles. These models also consider so-called hit-and-run scenarios, in which EBV initiates risk but does not remain as a persistent intrathecal antigenic stimulus [49]. In practice, primary prevention strategies, such as preventing EBV infection or modifying the outcomes of primary infection, and late-stage interventions, including targeting meningeal TLS-like inflammation and other CNS-compartmentalized processes, may require distinct intervention classes, target engagement strategies, and clinical endpoints [45]. These distinctions motivate treating EBV prevention as a conceptually separate lever from strategies aimed at established progressive biology, which are discussed next in the context of meningeal inflammation and CNS-compartmentalized therapeutic targeting [10].

### 3.4. Molecular Mimicry and Epitope Spreading

A mechanistic link between EBV and MS has been proposed to involve antigen-specific misdirection of adaptive immunity, in which antiviral responses may cross-recognize central nervous system proteins (molecular mimicry) and subsequently broaden as tissue injury progresses (epitope spreading) [50]. This framework may help explain why EBV infection is widespread while MS remains relatively rare, as only a subset of individuals may develop potentially pathogenic cross-reactive immune responses under specific host conditions [49]. Accordingly, molecular mimicry is best considered one possible mechanism within a broader EBV–MS framework, rather than a pathway that necessarily applies to all patients [50].

#### 3.4.1. Molecular Mimicry: Epstein–Barr Virus Epitopes with Cross-Reactivity to Central Nervous System Proteins

One of the most mechanistically developed examples involves EBNA1-directed immune responses that cross-react with the central nervous system protein GlialCAM (Glial Cell Adhesion Molecule). This finding is supported by evidence of clonally expanded B cells and functional binding assays, which together confirm genuine cross-reactivity. This matters because it demonstrates cross-reactivity at the level of antigen-specific clones rather than relying only on population-level correlations in antibody titers [51]. In a large human case–control study, antibody reactivity against EBNA1 and GlialCAM differentiated MS patients from controls, and inhibition experiments supported cross-reactivity rather than independent, unrelated antibody responses [52]. Even with this level of mechanistic support, an open question is whether such cross-reactive responses are typically initiating events or whether they are enriched and amplified once MS-related inflammation is already established [51]. Recent data also suggest that EBV-associated cross-reactivity may extend beyond a single antigen pair and may involve broader patterns of antiviral immunity intersecting with CNS-relevant targets [53]. Longitudinally anchored analyses have linked fine specificity to the EBNA1_381–452 region with cross-reactive antibody signatures against multiple CNS-related peptides around MS diagnosis compared with matched controls [54].

Previous studies demonstrate EBNA1 cross-reactivity with alpha-crystallin B (CRYAB), suggesting an additional mechanism through which EBV-directed humoral immunity may interact with central nervous system stress proteins during inflammation. Mechanistic evidence at the cellular level shows that EBNA1-primed CD4+ T cells can cross-recognize the MS-associated autoantigen anoctamin-2 (ANO2), and repertoire-level analyses reveal shared T-cell receptor features between EBV- and ANO2-reactive populations [55]. This T-cell dimension is notable given the established importance of antigen-specific T-cell programs in CNS entry, persistence, and inflammatory amplification in MS [56]. Taken together, these observations support the idea that EBV-directed immunity in MS may be altered not only in magnitude but also in antigen selection and cross-reactive breadth, yet uncertainty remains [38]. It is not yet resolved whether EBV-associated mimicry most often precedes CNS inflammation or becomes detectable because inflammation changes antigen access, trafficking, and immune selection pressures [56]. It is also unclear whether risk is dominated by a small number of higher-impact mimicry pairs or by the cumulative effect of many low-to-moderate cross-reactivities across individuals [50]. Consistent with this, cross-reactive immunoglobulin profiles reported in MS show substantial heterogeneity across patients [57].

#### 3.4.2. Epitope Spreading: Diversification of Autoimmune Specificity

A cross-reactive immune event can make the autoimmune response more diverse over time. This process, called epitope spreading, happens when injury in the central nervous system releases more antigens and keeps them present in inflamed areas. In MS, ongoing neuroinflammation allows the immune system to process and present antigens for longer, so the response can move from the original target to other parts of the same protein or to different CNS proteins [56]. In large serologic studies, antibody patterns that go beyond one cross-reactive target suggest that the immune response is diversifying. Some of these patterns also show signs of spreading around GlialCAM-related responses [52]. More generally, the wide variety of cross-reactive immunoglobulin profiles seen in MS supports the idea that antigen specificity can broaden over time, instead of focusing on one main autoantigen found in all patients [57]. However, demonstrating the temporal sequence of epitope spreading in humans is challenging. Such analysis requires frequent collection of preclinical samples and assays capable of monitoring changes in immune specificity within individual subjects [56]. Because of this, epitope spreading is best seen as a possible factor that increases MS-related autoimmunity after immune changes caused by EBV, rather than as the only reason for the link between EBV and MS [49].

## 4. Human Evidence Linking Epstein–Barr Virus to Multiple Sclerosis Risk

### 4.1. Serology and Temporality: Epstein–Barr Virus Exposure Preceding Multiple Sclerosis

**Figure 3:**
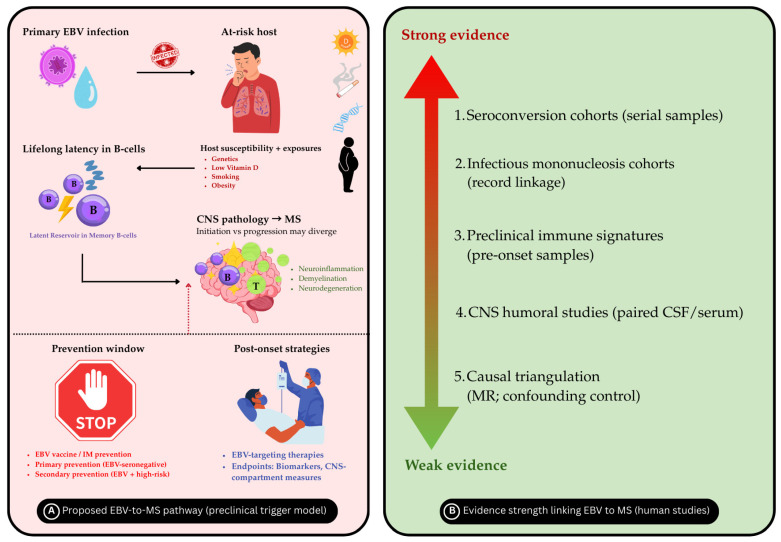
Conceptual EBV–MS pathway and relative strength of human evidence streams. (EBV, Epstein–Barr virus; MS, multiple sclerosis; CNS, central nervous system; CSF, cerebrospinal fluid; MR, Mendelian randomization).

The hypothesis that Epstein–Barr virus contributes to the development of MS is primarily based on the temporal relationship between exposure and onset of the disease. EBV infection must occur prior to the appearance of early biological changes and the onset of clinical symptoms [49]. Consistent with this logic, multiple independent studies have demonstrated that a heightened immune response to EBV, especially to EBV nuclear antigens, is detectable several years before the initial manifestation of MS symptoms. These findings argue against a purely post-diagnosis epiphenomenon [58]. The strongest evidence for temporality comes from a longitudinal study that analyzed over 10 million U.S. military personnel with serial serum samples collected over approximately 20 years. Among 955 individuals who developed MS during service, EBV seroconversion preceded MS onset in nearly all cases. MS occurrence among individuals who remained EBV-seronegative was exceedingly rare, supporting a strong temporal association between EBV infection and subsequent MS development. Importantly, although EBV seroprevalence in adults exceeds 90–95%, the overall lifetime risk of MS remains low (around 0.1–0.3%), indicating that EBV infection is likely necessary but not sufficient for disease development. In that same cohort, MS risk increased 32-fold after EBV seroconversion, while seroconversion to other viruses, including the similarly transmitted cytomegalovirus, did not show a comparable association. Importantly, serum neurofilament light chain levels increased only after EBV seroconversion, indicating that neuroaxonal injury occurred with infection rather than from pre-existing degeneration [16,34,59,60,61]. Analysis of preclinical samples from Sweden indicates that EBNA1 seroreactivity increases as early as 15 to 20 years prior to the onset of clinical MS. Subsequently, lytic antigen seroreactivity, including gp350, also rises. Within the same cohort, serum neurofilament light levels increased on average nearly a decade after the EBV-associated changes, suggesting a prolonged interval between immune alterations and detectable neural injury [59]. Detailed analysis of the EBV peptidome in pre-onset blood samples suggests that people who later develop MS have a stronger overall antibody response to EBV. However, there is no single MS-specific peptide that clearly distinguishes cases from controls when total anti-EBNA1 reactivity is considered [58]. This finding indicates EBV-directed immunity can become dysregulated quantitatively. Researchers can measure and track this by looking for persistent, high-titer responses to defined EBNA1 regions after EBV seroconversion. Longitudinal sampling anchored to EBV seroconversion demonstrated that elevations in EBNA-1_ {381–452}-specific IgG were detectable as early as nine months post-seroconversion and a median of 5.4 years prior to MS diagnosis in that study [60]. A related point of negative evidence is that rigorously adjudicated MS is uncommon among EBV-seronegative pediatric inflammatory demyelination cases. In these instances, EBV-seronegativity should prompt careful consideration of alternative diagnoses, such as myelin oligodendrocyte glycoprotein antibody disease [61]. An important limitation is that serology indicates exposure and host immune response rather than direct localization of viral activity within central nervous system compartments. Therefore, while temporality is robust, serology alone does not provide a tissue-level mechanism readout. Generally, taken together, the serologic timeline indicates that EBV infection precedes the amplification of EBV-directed immune signatures over several years, followed by the subsequent emergence of measurable neuroaxonal injury. This sequence is difficult to explain by reverse causality alone (Figure 3) [34].

Table 2 summarizes the main streams of evidence linking EBV to MS. It outlines the type of study design, the key inference supported by each evidence stream, and its primary limitations. Together, these complementary approaches provide temporal, epidemiological, immunological, and genetic perspectives, while also defining the boundaries and uncertainties that remain in causal interpretation.

### 4.2. Infectious Mononucleosis and Risk Modification: A Natural Experiment for Immune Intensity

Infectious mononucleosis represents the clinical syndrome of symptomatic primary EBV infection, which occurs more often when first exposure is delayed to adolescence or early adulthood rather than early childhood [17]. This distinction is of relevance since MS risk appears to be more strongly associated with IM rather than with EBV infection per se, suggesting that the host response during primary infection may modify later autoimmune risk [70]. Population-level analyses consistently demonstrate that IM is associated with an increased subsequent risk of MS, with pooled estimates indicating that IM more than doubles the risk of MS. A significant advantage of recent studies is the utilization of routinely collected health records, which reduces recall bias associated with questionnaire-based case–control ascertainment of prior IM. In an English national hospital record cohort spanning 2003–2023, restricted to individuals younger than 25 years to preserve temporality, MS incidence was nearly three times higher after hospital-diagnosed IM than in a large reference cohort, after multivariable adjustment. The same study found minimal evidence of an increased incidence of MS within the first four years following infectious mononucleosis. However, hazard ratios peaked five to nine years post-IM and remained elevated for up to 10 to 20 years. This temporal pattern indicates the presence of a prolonged preclinical interval rather than a short-term diagnostic bias [17]. Complementary evidence from a large German outpatient cohort also reported increased incident MS diagnoses following IM, supporting the reproducibility of this association across healthcare settings and data sources [35]. From a prevention standpoint, the IM signal is particularly valuable because it serves as a natural experiment that identifies delayed primary infection and heightened immune activation in real-world populations. IM also represents a practical intermediate target for interventions, as preventing symptomatic primary infection may be a more achievable vaccine goal than eliminating EBV infection in the population entirely [70]. Beyond IM alone, recent work supports a risk-stacking model, in which IM interacts with other exposures that potentially impair EBV immune control or promote neuroinflammation [62]. Analysis of a large Swedish population-based case–control dataset also indicated that a history of infectious mononucleosis was associated with an increased risk of MS. Additionally, several lifestyle exposures demonstrated synergistic interaction signals with IM status on the additive scale. Specifically, the study found that individuals with prior IM had an odds ratio of 1.86 for developing MS compared to those without IM. It also identified significant interactions with exposures such as passive smoking, alcohol and fish consumption, vitamin D status, and adolescent sleep patterns [63]. Collectively, the IM literature indicates that EBV-related risk is not strictly binary (infected versus not infected) but is influenced by the clinical phenotype of primary infection and by co-exposures that may alter immune regulation over time [62].

### 4.3. Epstein–Barr Virus Immune Signatures in Preclinical and Early Multiple Sclerosis

In addition to EBV seropositivity, several studies have reported that individuals who later develop MS show differences in immune responses to EBV, both in magnitude and pattern. These observations suggest altered immune regulation or antigen-specific responses preceding clinical onset [71]. Peptidome analyses of presymptomatic samples have shown stronger antibody responses to EBV in individuals who later developed MS compared with controls, particularly against EBNA1. However, no single EBV peptide consistently distinguished cases from controls, indicating a broader shift in immune reactivity rather than a single dominant target [58].

Longitudinal studies have further shown that seroreactivity to EBV antigens, including both latent and lytic proteins, can be detected years before clinical disease, in some cases preceding markers of neuroaxonal injury [59]. In one study, higher EBNA1-specific IgG levels were observed after seroconversion in individuals who later developed MS and remained elevated over time, suggesting that sustained humoral responses may be associated with increased risk [60].

Additional serological studies have found higher antibody responses to multiple EBV antigens in people with MS compared with EBV-seropositive controls, although evidence for a major role of ongoing viral reactivation is limited. Signals suggestive of molecular mimicry have been reported for some targets, but findings have not been consistent across studies [72].

When considering CNS compartments, analyses of paired cerebrospinal fluid and serum samples indicate that intrathecal EBV-specific antibody production is relatively uncommon compared with responses to other pathogens. This suggests that EBV may contribute more to immune priming or modulation than to a dominant intrathecal antiviral response [65,66].

### 4.4. Confounders and Alternative Explanations: Genetics, Environment, and Reverse Causality

Because EBV infection is nearly universal, studies of MS often focus on differences in the timing and clinical presentation of infection, such as delayed infection or infectious mononucleosis (IM). These factors may be linked to socioeconomic conditions and patterns of social contact, although such variables are not always well captured in health records. As a result, residual confounding may partly influence observed associations, particularly in studies using IM as a marker of delayed infection or stronger immune response [73].

Host genetics also plays an important role, as MS susceptibility is strongly influenced by immune-related loci. Genetic background may affect both EBV immune control and the likelihood of symptomatic infection. In this context, EBV-related immune responses may reflect interactions between genetic and environmental factors rather than independent effects. Environmental exposures such as smoking and low vitamin D levels are established MS risk factors and may also influence immune regulation relevant to EBV [62]. Integrative models therefore consider EBV within a broader network of interacting influences, including immune and environmental factors [74].

Reverse causality has been proposed as an alternative explanation, suggesting that early, preclinical immune changes could increase susceptibility to symptomatic EBV infection or higher antibody levels. However, longitudinal studies with repeated sampling suggest that key biomarkers, such as serum neurofilament light chain, increase after EBV seroconversion, supporting a temporal sequence in which infection precedes disease-related changes [16]. Similarly, early differences in EBNA1-specific antibody responses have been observed shortly after seroconversion in individuals who later develop MS [60].

In studies based on IM, diagnostic and surveillance bias are also considerations, as increased healthcare contact may lead to earlier MS detection. However, the persistence of elevated risk over time argues against this being solely due to short-term detection effects [17]. Genetic approaches, such as polygenic scoring and Mendelian randomization, can help address confounding and reverse causation, although their interpretation depends on underlying assumptions and is strengthened when supported by multiple lines of evidence [67].

## 5. Prevention and Interception Strategies Targeting Epstein–Barr Virus

### 5.1. Prophylactic Epstein–Barr Virus Vaccines

#### 5.1.1. Platform Strategies (mRNA, Protein Subunit, Vector, and Others)

Most prophylactic EBV vaccine concepts aim to interrupt the infection in the earliest steps, since EBV establishes long-lived latency soon after primary exposure and controlling it later on is difficult [75]. A major challenge is EBV’s dual tropism, which allows the virus to infect both B lymphocytes and epithelial cells, and it uses several glycoprotein complexes to enter. This means vaccines targeting only one viral protein may not provide full protection [76]. mRNA platforms are attractive for EBV because they can encode several envelope glycoproteins in a single formulation, enabling multivalent designs intended to cover different entry pathways [77]. The clinical mRNA candidate mRNA-1189 is currently under evaluation in a Phase 1 study involving healthy adolescents and adults, with primary objectives assessing safety, reactogenicity, and immunogenicity [78]. Protein-based strategies continue to advance, with nanoparticle display approaches aiming to enhance antigen presentation and increase neutralizing antibody responses compared with soluble subunit immunogens [75]. A clinical example is the NIH-led evaluation of an adjuvanted EBV gp350–ferritin nanoparticle vaccine. This high-density display, combined with a potent adjuvant, is intended to induce robust humoral responses against a well-characterized EBV attachment antigen [79]. Early-Phase 1 results reported that the gp350–ferritin nanoparticle vaccine, adjuvanted with Matrix-M1, was safe, well-tolerated, and immunogenic in a first-in-human, open-label setting [80]. Preclinical vaccine development is now focusing more on combining glycoproteins, such as gH/gL, gB, and gp42. This approach aims to improve neutralization across different cell types and lower the risk of breakthrough infections, even when B-cell-targeted antibodies are present [81]. Researchers are also exploring replicon RNA strategies, in addition to traditional protein and mRNA methods. These strategies may boost antigen expression and help strengthen both humoral and cellular immunity in non-infectious vaccine designs [82].

#### 5.1.2. Endpoints for Efficacy (Infection Prevention, Infectious Mononucleosis Prevention, and Reservoir Modification)

One potential strategy for preventing MS is to prevent initial infection EBV. In theory, this could avoid the establishment of viral latency and remove EBV from the disease pathway. However, achieving complete prevention of infection is challenging, as EBV can enter the host through multiple routes and establish latency early. As a result, partial prevention of viral entry may still allow asymptomatic infection [18].

A more practical near-term goal may be the prevention of infectious mononucleosis (IM), which is associated with an increased risk of MS in population-based studies [17]. This endpoint may be more achievable in the context of vaccine development, as reducing symptomatic infection does not require complete prevention of EBV acquisition and may still be clinically meaningful while long-term MS outcomes remain difficult to assess [83]. Supporting this distinction, vaccine studies targeting gp350 have shown a reduced incidence of IM without achieving sterilizing immunity, suggesting that prevention of symptomatic disease and prevention of infection may represent different outcomes [81]. Another possible approach is to modify the host–virus balance after infection, for example, by reducing the size or activity of the latent EBV reservoir. This could potentially influence MS risk even if infection is not fully prevented [18]. However, assessing such effects is challenging, as direct measurement of latent reservoirs in relevant tissues is not feasible at scale. Therefore, studies may rely on indirect markers, such as viral shedding, immune responses, or long-term changes in blood-based measures, rather than definitive endpoints such as elimination of latency [83].

#### 5.1.3. Correlates of Protection (Neutralizing Antibodies and Cellular Immunity)

The most actionable correlate candidates remain neutralizing antibodies targeting EBV envelope glycoproteins, as entry blockade can be quantified in cell-based assays and directly reflects early infection biology [18]. However, identifying correlates is complicated by EBV’s distinct entry mechanisms into B cells and epithelial cells. This complexity necessitates the measurement of neutralization in both cellular contexts rather than relying on a single assay format [76]. Preclinical studies of multivalent vaccine designs demonstrate that incorporating gH/gL- and gB-targeting components broadens neutralization compared to gp350-focused immunity alone. These findings support the rationale for entry-complex-informed antigen selection [81]. Cellular immunity is also likely to be important for long-term control if vaccines do not fully prevent infection. T-cell surveillance is central to containing EBV-infected B cells and may influence the stability and behavior of the latent reservoir [82]. From a development perspective, correlates will need to be scalable and harmonized across studies. Standardized neutralization panels and reproducible serologic readouts are essential for comparing platforms and selecting clinical endpoints [75].

### 5.2. Antivirals: What They Can and Cannot Do for Latency

Most licensed anti-herpesvirus drugs cause inhibition of viral DNA replication during the lytic phase of infection. As a result, these agents suppress lytic EBV replication but fail to eliminate latent EBV genomes that persist in long-lived memory B cells. The inability to target both lytic and latent EBV infection is a major limitation of current drug strategies for MS prevention. Most MS-related EBV hypotheses focus on virus persistence and its immunological effects during latency, rather than on ongoing lytic infection [84]. As a result, recent MS-focused antiviral studies have measured EBV shedding to determine whether standard antiviral agents can alter EBV activity within a feasible clinical trial timeframe. In a pilot study, famciclovir did not significantly reduce salivary EBV shedding in individuals receiving natalizumab. This finding highlights the difficulty of modifying EBV biology with agents that exclusively target the lytic phase in this context [85]. These results do not preclude the use of antiviral therapies; however, they indicate that effective prevention may require either intervention at the time of primary infection or the development of novel agents capable of directly targeting latent infection [84].

### 5.3. Immunotherapies as Epstein–Barr Virus Mechanism Modifiers

#### 5.3.1. Epstein–Barr Virus-Specific T-Cell Approaches

Given the central role of T-cell immune monitoring in the long-term containment of EBV-infected cells, EBV-specific cellular immunotherapies offer a direct mechanistic approach to improving EBV control [84]. An allogeneic EBV-specific T-cell immunotherapy designed to target EBV-infected cells in an HLA-restricted manner has been described [86]. In MS, early-phase studies of autologous EBV-specific T-cell therapy for progressive disease have demonstrated acceptable safety and indicated sustained clinical improvement in a subset of patients. However, the current evidence base is limited and heterogeneous [87]. For prevention or preclinical intervention, the risk–benefit threshold is considerably higher than in progressive disease. Therefore, EBV-specific immunotherapy is more appropriately positioned as a tertiary or mechanistic strategy rather than as a population-level prophylactic measure [84].

#### 5.3.2. B-Cell-Targeted Strategies and the Specificity Problem

Because EBV latency mainly occurs within B-cell compartments, therapies targeting B cells are often considered indirect approaches to modulate EBV, even when they do not directly target the virus. Although this approach is theoretically attractive, it presents clinical limitations. Broad B-cell targeting may induce immunosuppression, which is difficult to justify for disease prevention in otherwise healthy individuals [84]. Therefore, if B-cell-directed strategies are considered for EBV, their most appropriate application is in secondary prevention among individuals at elevated risk, such as those with RIS or CIS, where the likelihood of disease progression may warrant more intensive interventions [8].

### 5.4. Secondary Prevention: Interception Trials

Intercept trials aim to prevent or delay clinical conversion by targeting preclinical or early clinical stages, which are higher risk and have shorter follow-up periods than in traditional primary prevention studies [8]. Radiologically isolated syndrome is now seen as a practical stage for interception, since new diagnostic criteria and recent data show a measurable risk of a first clinical demyelinating event. This makes it possible to run smaller, more practical trials than those with EBV-seronegative groups [88]. Several interception trials are currently evaluating whether early administration of disease-modifying therapy can prevent or delay conversion in RIS. These studies demonstrate that the concept of a preclinical MS window is now practically relevant in clinical research [89]. Trial populations can be further refined by including EBV-related immune markers, such as persistently elevated EBNA-1(381–452)-specific immunoglobulin G levels after seroconversion. These biomarkers have been linked to subsequent MS diagnosis years before the onset of symptoms [60]. An additional enhancement strategy involves using prior infectious mononucleosis as a proxy for delayed symptomatic primary EBV infection. This approach is associated with a significantly elevated risk of MS development in long-term population studies [17]. Research on interception of radiologically isolated syndrome and clinically isolated syndrome can use both time-to-event outcomes and standardized MRI activity measures, as well as scalable blood biomarkers, as endpoints [8]. Blood biomarkers offer sensitive indicators of neuroaxonal injury and inflammatory activity and are therefore central to interception strategies. They complement imaging, particularly in cohorts with minimal symptom burden [90]. Advanced imaging markers, including the central vein sign, volumetric analysis, and connectivity measures, may further refine risk stratification. These tools can reduce required sample sizes by identifying individuals with a higher probability of near-term conversion [91]. At the moment, digital assessments are being evaluated as adaptable tools for detecting subtle functional changes and enhancing participant involvement. These approaches may improve options for preclinical populations, where standard disability measures are often not sensitive enough [92]. Clinical feasibility is further supported by the development of multicenter collaborations. For example, the European Committee for Treatment and Research in Multiple Sclerosis (ETRIMS) has endorsed RIS to standardize study endpoints and sample collection across research sites [93].

Despite promising conceptual approaches, the real-world implementation of EBV-targeted prevention strategies faces important challenges. These include the high prevalence of EBV infection, the long time between infection and MS onset, and the difficulty in identifying individuals at sufficiently high risk for intervention. In addition, measuring meaningful clinical outcomes in prevention studies requires long follow-up and large cohorts, which may limit feasibility. Therefore, while EBV-targeted strategies are scientifically attractive, their translation into clinical practice will require carefully designed trials, improved risk stratification, and realistic expectations regarding achievable endpoints.

## 6. Biomarkers and Enrichment Tools to Make Prevention Testable

Prevention and detection trials for MS will only be feasible if the risk can be defined with measurable enrichment tools, because the baseline incidence of MS is low and the latency between exposure and clinical onset can span years [94]. The most useful biomarkers in an EBV-focused prevention framework are those that identify individuals in a biologically plausible preclinical window or provide intermediate endpoints that change sooner than clinical diagnosis [95].

### 6.1. Serologic Signatures

Due to the high prevalence of EBV infection, serological assessments in prevention studies primarily aim to quantify humoral response patterns to differentiate MS cases from EBV-positive controls, rather than to exclusively confirm exposure. A large peptidome-based case–control study identified EBNA-1-directed responses as the most robust serologic signal associated with subsequent MS risk, thereby supporting the use of EBNA-1-centric assays as candidate enrichment tools [58]. A longitudinal approach anchored to individual EBV seroconversion demonstrated that EBNA-1(381–452)-specific IgG titers were elevated early after seroconversion in individuals who subsequently developed MS. This finding suggests that informative humoral divergence may arise soon after EBV acquisition [60]. Serologic signatures can be considered alongside CSF measures, as intrathecal antibody patterns provide additional information about immune activity in specific areas. This approach may help identify early MS cases for interception studies. In this setting, intrathecal EBV-directed IgG production is less common than polyspecific intrathecal responses to other microbial antigens. This suggests that, for most patients, it is unlikely that there is simply ongoing EBV reactivation in the CSF [65]. Moreover, antibody titers are not direct readouts of EBV reactivation, and single-time-point interpretation can be confounded by long-lived antibody persistence and physiological fluctuation. Therefore, serology is best treated as an enrichment signal rather than a standalone mechanistic endpoint [15].

### 6.2. Cellular Immune Profiling

Cellular profiling is useful for EBV-driven prevention ideas because long-term control of EBV relies on antigen-specific T-cell surveillance. Several studies have found changes in EBV-directed cellular immunity in MS. For trials, the most convenient cellular readouts are those that can be standardized across different sites. These include antigen-specific cytokine responses, activation or exhaustion markers, and repertoire-level features, instead of custom assays that are hard to reproduce [38]. In addition to the usual EBV-specific CD8 and CD4 responses, other immune cell types, such as CD20+ T cells, have been linked to early MS inflammation. These cells may act as both markers and possible targets in early-stage groups [96].

### 6.3. Viral Load and Reactivation Proxies (And Limitations)

Tests such as EBV DNA detection by PCR, serologic reactivation patterns, and shedding proxies are commonly used because they are easily accessible. However, these assays do not directly reflect EBV lifecycle states in the tissue compartments most relevant to MS; therefore, their results should be interpreted with caution [15]. The reliability and interpretation of assays depend on specimen type, analytic thresholds, and clinical context. Low EBV DNA levels may indicate latent carriage rather than active lytic replication [97]. In prevention trials, these measures are best used as supportive biomarkers for stratification, covariate adjustment, or mechanistic exploration, rather than as primary endpoints to demonstrate changes in latent reservoir biology [15].

### 6.4. Multi-Omic Integration and Artificial Intelligence-Assisted Risk Models

The primary justification for artificial intelligence (AI)-assisted enrichment emerges when models integrate modalities with established biological relevance in MS. Multimodal fusion enhances discrimination and reduces dependence on individual noisy markers [98]. An example of clinically interpretable multimodal stratification is the combination of imaging features with serum neurofilament light chain. This method has identified biologically distinct MS subtypes and demonstrates how scalable blood markers can be combined with MRI to improve prognostic resolution [99]. In the context of prevention, the most relevant near-term application of AI is not black-box diagnosis. Instead, it entails constructing validated risk enrichment layers that integrate EBV immune signatures with established MS biomarkers and imaging features in a manner that is portable across cohorts [100]. Given that dataset shift and overfitting are constant challenges in clinical machine learning (ML), any model intended for trial enrollment should undergo external validation, calibration, and, ideally, prospective testing before being used to define high-risk status for preventive interventions [98].

### 6.5. Practical Enrichment Hierarchy for Trial Readiness

One effective way to use these tools is to apply a step-by-step enrichment strategy. First, use broad markers, such as EBV serologic signatures and a history of infectious mononucleosis, to identify an initial group at risk. Then, add more specific tests, including MRI features, serum neurofilament light chain, and standardized cellular immune profiling, to focus on those with higher near-term event risk and improve trial efficiency [83].

Taken together, these approaches suggest a practical way to make EBV-related prevention studies in MS possible. Instead of relying on one marker, a step-by-step approach is needed, starting with serologic signals and adding cellular, imaging, and clinical markers to better identify people at higher risk. Serology can help with early selection, while other tools improve accuracy and study design. At the same time, there are important limits, especially because many biomarkers are indirect and not fully validated. This framework helps connect biological ideas with clinical use, but further research is still needed.

## 7. Clinical Trial Roadmap and Implementation Challenges

### 7.1. Primary Prevention Trial Designs (Epstein–Barr Virus-Seronegative Cohorts)

Ideally, true primary prevention trials would enroll EBV-seronegative patients before the usual age of acquiring the virus and randomly assign them to receive either the vaccine or a control. The main short-term goals would be to see if the vaccine prevents EBV infection and infectious mononucleosis. Since MS often appears many years after EBV exposure and is relatively rare, preventing MS is not a practical main goal for early vaccine trials. A more realistic approach is to proceed in stages: first, early studies establish safety and immune response; next, later trials evaluate whether the vaccine protects against important EBV-related illnesses. Finally, long-term follow-ups, supported by health records or registries and, when possible, collecting samples over time, can assess whether MS rates differ between vaccinated and unvaccinated groups [73].

### 7.2. Secondary Prevention Designs (High-Risk Epstein–Barr Virus-Positive Cohorts)

Secondary prevention (interception) trials are easier to run in EBV-positive individuals who are near the onset of clinical disease. These groups have higher event rates and need shorter follow-up, making studies more practical. Radiologically isolated syndrome is a useful model for these trials because new criteria help identify people without symptoms who are at risk for a first demyelinating event. These criteria also allow researchers to sort risk levels using MRI and CSF results [88]. Researchers can use a stepwise enrichment approach: first, using RIS or CIS definitions and MRI risk features; second, looking for signs of inflammation in the spinal fluid, such as immunoglobulin indices or oligoclonal bands; and third, focusing on people with high levels of EBV antibodies after seroconversion. This procedure helps select those most likely to develop symptoms soon. Research in other groups confirms that the 2023 criteria are good at predicting risk, and adding CSF immunoglobulin indices can further improve accuracy, which is important when treatments carry immune risks [101]. Existing RIS trials provide operational examples of feasible endpoints for interception studies, including time-to-first clinical event and MRI-based activity measures, with biomarker and cognitive substudies as secondary or exploratory outcomes. This design framework can be adapted for EBV-targeted interception by selecting endpoints that capture both clinical conversion and subclinical injury, while ensuring practical sample sizes and follow-up durations [102].

### 7.3. Tertiary Mechanistic Trials in Established Multiple Sclerosis

In cases of established MS, especially in progressive forms, EBV-targeted studies are best used as mechanistic or target engagement trials. The main goal is to establish whether intervention changes EBV-related immune networks or compartmentalized inflammation, rather than to demonstrate prevention. This approach also helps clarify which endpoints to use. Traditional lesion metrics often miss CNS-compartmentalized activity, so trials now focus more on measures that track chronic active lesions, signs of meningeal inflammation, and biomarker changes that better show progression-related disease [45]. Scalable fluid biomarkers also help researchers study disease mechanisms by measuring neuroaxonal injury and glial activation over shorter timeframes than traditional disability measures. A major advantage is that these biomarker panels can be used in both secondary prevention and mechanistic trials, with standardized tests. This makes it easier to compare biological effects across studies, even when clinical endpoints differ [90].

### 7.4. Safety, Ethics, and Long Follow-Up Horizons

As clinical trials begin to include healthy adolescents, ethical standards are enforced because acceptable risk is much lower, and there is less tolerance for uncertainty, especially for treatments that affect the immune system in new ways. These factors shape how trials are designed, with a focus on spotting safety issues early, using strict rules for stopping the trial, and carefully choosing surrogate endpoints. Trials also need strong long-term safety monitoring plans that continue to work as participants move from pediatric to adult healthcare [103]. Human challenge frameworks are often seen as a means to accelerate vaccine development for infectious diseases; however, these models pose additional ethical and oversight challenges, particularly when long-term effects are uncertain or when challenge agents and endpoints lack standardization. Recent regulatory discussions emphasize the importance of aligning protocols, setting clear goals, and ensuring strong oversight to balance research benefits with individual safety and burden. Consequently, applying such models to EBV would require particularly strong justification [104].

### 7.5. Regulatory and Public Health Considerations

From a regulatory standpoint, vaccine development should follow the pathway of first checking safety and immune response, then testing efficacy. Some studies also examine immune markers that indicate protection or effectiveness, which can help inform labeling and public health advice. The European Medicines Agency (EMA) says it is important to consider clinical results, immune response, effectiveness, and protection markers when evaluating vaccines. This is especially important for EBV vaccines, if the first approved use is to prevent infectious mononucleosis rather than to prevent infection completely [105]. Even if an EBV vaccine receives licensure based on infection-related endpoints, justifying a credible claim for MS prevention will likely entail robust post-licensure effectiveness infrastructure. This approach embraces linkage-capable cohorts, standardized case definitions, and impartial access patterns intended to reduce the risk of vaccine uptake being confounded by economic and social factors and its association with MS risk. This public health consideration is essential, as the evidentiary standard for demonstrating EBV prevention differs from that required for MS prevention. The latter will likely require integrating trial data, long-term surveillance, and mechanistic biomarkers that demonstrate a sustained modification of the EBV–host relationship [83].

## 8. Future Directions

Although considerable epidemiological and mechanistic evidence has accumulated to support the critical role of EBV in the pathogenesis of MS, some critical issues remain to be resolved before the application of anti-EBV strategies can be considered for prevention or treatment of the disease [106]. One of the critical issues is the lack of well-established causal correlates that can be used to differentiate between ubiquitous exposure to EBV and pathogenic virus–host interactions that result in the development of MS. Indeed, since over 90% of the adult population is seropositive for EBV, whereas only a small percentage of this population is at risk of developing MS. Seropositivity is obviously not sufficient as a correlate of risk for developing the disease [49]. Future studies should move beyond the simple exposure–pathogenesis model and instead define quantitative (e.g., viral load, latency/reactivation, EBV burden in memory B cells) and qualitative (e.g., T-cell exhaustion, impaired cytotoxicity, cross-reactive antibodies) correlates of risk [38,107]. Equally important is the incorporation of host susceptibility factors, such as HLA types, epigenetic modifications, and immune checkpoint molecules, into predictive models that allow for stratification based on biologically relevant risk for MS [108]. A major barrier to translating these advances into practice is the lack of standardized and scalable assays for EBV surveillance. The majority of existing studies employ research-grade surrogate markers of EBV infection, peripheral blood EBV load analysis, or sophisticated immune response analysis tools that are not readily available for routine clinical practice or population surveillance [15,64]. In the absence of robust, cost-effective, and standardized diagnostic tools for EBV and immune response surveillance, it will not be possible to operationalize EBV risk stratification or preventive measures in routine healthcare settings. The development of such tools is therefore a prerequisite for any credible preventive model [15,64]. Finally, the available cohort infrastructure is currently inadequate for addressing the temporal and causal complexity of the interactions between EBV and MS. The best available evidence to date has been obtained from large retrospective or semi-prospective cohorts. However, these approaches are inherently limited in the potential to inform early immune system interactions, virus dynamics, and preclinical neuroinflammatory processes [16,49]. Clearly, the way forward in this field will require the establishment of cohorts that are specifically designed and prospective in nature and, that begin in childhood or adolescence, and that include virological and immunological profiling and risk stratification according to genetic risk [109,110]. From the point of view of clinical trial design, this infrastructure is essential because any attempt to prove the prevention of MS will require extremely long follow-up periods, extremely large cohorts, and the use of surrogate endpoints long before the onset of clinical disease. Without such cohorts, the prevention of EBV will be an epidemiologically compelling but clinically unachievable goal [8,111,112]. The key challenge is defining the level of evidence needed to credibly show that preventing EBV infection can prevent MS. The multifactorial and long-latency process of MS pathogenesis means that the level of evidence needed would have to exceed that required to support the presence of conventional associative risk factors by a considerable margin [49,50]. The level of evidence needed would have to approximate that required to support disease-modifying interventions. At the epidemiological level, any EBV prevention strategy that is implemented through vaccination, antiviral agents, or immunomodulation would have to demonstrate not only that EBV infection is being suppressed or prevented but also that there is a statistically significant and clinically important reduction in MS risk compared with appropriate control populations [70,83]. The effects would have to be reproducible in both the general population and high-risk populations that are genetically or immunologically predisposed to MS. Ideally, the effects would have to demonstrate a dose–response relationship between the degree of viral suppression and MS risk. Without this convergence, reduced MS incidence could reflect indirect or confounded effects rather than true causal prevention [36,68]. Nevertheless, epidemiological divergence alone would be insufficient to support a causal inference for a disease as multifaceted as MS. A legitimate causal hypothesis would need to be supported by parallel mechanistic evidence that shows that prevention of EBV results in biologically meaningful changes in well-established immunopathological pathways in the disease, such as alterations in the immune microenvironment of the CNS, reduction in autoreactive or cross-reactive B-cell clones, diminution of ectopic lymphoid follicle formation in the meninges, and normalization of critical proinflammatory signaling cascades involving Th17 cells, microglial activation, and BBB permeability [52,113,114]. From a clinical trial perspective, the theoretical gold standard would be to obtain results from a randomized, long-term EBV prevention study that shows decreased MS incidence. However, the logistical, ethical, and temporal challenges of conducting such a trial make it essentially impossible [112]. As such, the minimum evidence framework that is likely necessary to support the efficacy of EBV prevention will necessarily be a composite of indirect but converging approaches, including vaccine or antiviral trials that have shown efficacy of EBV infection or latency prevention, chronic divergence of MS incidence in epidemiological studies, and supportive causal inference from Mendelian randomization and mediation approaches [69,83]. Only by considering these approaches collectively will it be possible to consider EBV prevention as more than simply a plausible but unproven approach to disease modification. Even if the role of EBV in MS is conclusively proven, the application of this knowledge in the real world would also present many practical, ethical, and health policy problems. Perhaps the first problem to be solved would be the identification of the target population for intervention [115]. If EBV vaccination or antiviral therapy is to be used to prevent MS, this would mean treating many healthy people with prolonged interventions to prevent a relatively rare disease, which would also raise important questions of cost-effectiveness, balance of risks and benefits, and public health resource allocation. On the other hand, focusing on those at genetic or immunological risk of developing MS would require the availability of tools for stratifying risk that do not yet exist, while at the same time risking failure to capture a large percentage of future cases of MS that do not fall within the current defined high-risk groups [83,116,117]. The timing of the intervention is an equally crucial issue. Primary prevention prior to EBV infection would be conceptually the most attractive option, although it would be limited by the early age at which EBV is acquired and the difficulty of implementing a widespread pediatric vaccination program against a disease that becomes clinically apparent many decades later [73]. Secondary prevention after the primary EBV infection and before the appearance of neurological symptoms would require the availability of sensitive biomarkers that can detect the presence of a preclinical autoimmune phenomenon, a phase that is still not well characterized in the clinical setting. Tertiary prevention after the first demyelinating event or the RIS might be the most accessible option, although the interventions against EBV might not be able to counteract the already established immunopathological events and might be limited to the modulation rather than the prevention of the disease [39,118]. This is further complicated by the modality of the therapeutic intervention. For example, the safety profiles of prophylactic vaccines, therapeutic vaccines, antiviral drugs for latent infection, and adoptive T-cell therapies are quite distinct from each other. Although vaccine-based strategies may be effective for prophylaxis, they may be inadequate for the clearance of the latent pool of EBV. Antiviral or immune-based therapies may be more effective, but may require long-term consequences of immunomodulation. These considerations highlight the fact that EBV prevention is a spectrum of possible strategies with distinct trade-offs for each modality [119,120,121]. From a health system perspective, the implication of widespread EBV-targeted prevention would be the need to rethink the way MS care is currently managed, from a reactive model of disease management towards long-term risk surveillance. This would involve screening programs, the integration of virology and immunology into primary care settings, and the long-term surveillance of asymptomatic individuals. Such an approach has obvious logistical and economic consequences. However, without considering the practicalities of such an approach, the feasibility of EBV prevention may be compromised and seen as scientifically compelling but practically impossible [112]. As outlined in the trigger–necessary–permissive framework described above, the central unresolved issue is whether EBV occupies a prerequisite position in MS pathogenesis or functions as a risk-modifying cofactor. The necessary cause model supports the implementation of active population prevention strategies, while the permissive cause model indicates that there are declining returns for universal prevention strategies and that the focus should be on multifactorial risk factor modification. The current data, although strongly suggestive of a causal relationship, do not fully support this hierarchy, as the majority of available data are indirect, observational, and subject to confounding. In addition, the existence of EBV-seronegative MS cases, although rare, has significant implications for the necessary cause model and the possibility of alternate or additional mechanisms of disease [17,34,62,122]. From a conceptual point of view, there is also a risk of oversimplifying the MS disease process as a virus-induced phenomenon, which might lead to an underestimation of the role of general immune dysregulation, genetic factors, microbiome interactions, and systemic inflammatory processes [108]. Although the EBV hypothesis unifies all the different aspects of the disease, including epidemiological, immunological, and molecular findings, it might represent only one level in a much more complex disease network. EBV might be considered a drug target, and this might lead to a reductionist approach that overlooks the adaptive and self-sustaining properties of autoimmune pathology [9,123]. However, the biggest challenge for the field in the long term is to differentiate between biological causality and clinical sufficiency. Even if EBV is proven to be a causal trigger, it does not necessarily follow that EBV-targeting interventions will be clinically effective in terms of prevention and disease modification. Without resolving this dichotomy, EBV prevention could be a biologically elegant but clinically incomplete solution to a fundamentally multifactorial disease [62,122].

## 9. Search Strategy

### 9.1. Literature Search Strategy

A structured literature search was conducted to identify relevant studies on the association between EBV and multiple sclerosis MS. Searches were performed in PubMed and Web of Science for articles published up to February 2026, using combinations of the following keywords: “Epstein–Barr virus”, “EBV”, “multiple sclerosis”, “neuroinflammation”, “risk factors”, and “prevention”. In addition, reference lists of relevant articles were manually screened to identify further eligible studies.

### 9.2. Study Selection and Eligibility

Studies were screened based on titles and abstracts, followed by full-text evaluation for eligibility. Eligible studies included original research articles, longitudinal cohort studies, and relevant reviews addressing EBV–MS associations, with particular emphasis on epidemiological, immunological, mechanistic, and prevention-related aspects. Exclusion criteria included non-English publications, case reports, and studies not directly relevant to the topic. Study selection was guided by relevance to the research question and conceptual contribution.

### 9.3. Data Synthesis and Methodological Approach

This review was designed as a narrative synthesis to integrate heterogeneous evidence across epidemiological, immunological, and mechanistic domains. Given the substantial heterogeneity in study designs, outcomes, and methodological approaches, a quantitative meta-analysis was not feasible. No formal protocol registration or standardized risk-of-bias assessment was performed. Instead, potential sources of bias, including study design limitations and heterogeneity, were considered qualitatively during data interpretation. The aim was to provide an integrative and conceptually grounded synthesis of current evidence, identify key areas of consistency and uncertainty, and highlight implications for prevention strategies.

## 10. Limitations

This review has several limitations. As a narrative review, no formal risk-of-bias assessment or protocol registration was performed, and no quantitative synthesis was undertaken. Although a structured search strategy and critical appraisal were applied, study selection and interpretation remain subject to potential bias, and the absence of standardized assessment tools may limit reproducibility and comparability. The included studies are heterogeneous in design, methodology, and quality, which may affect the consistency and interpretation of findings. In addition, most evidence linking EBV to MS is observational, limiting causal inference. Mechanistic understanding remains incomplete, particularly regarding the role of EBV within central nervous system compartments. Finally, generalizability may be influenced by geographic and population differences in virus exposure and disease risk.

## 11. Conclusions

MS is a chronic inflammatory disease of the central nervous system that often begins early in life and may lead to irreversible disability. Although current disease-modifying therapies reduce inflammatory activity, they do not reliably prevent progression or repair established damage, highlighting the need for preventive and early intervention strategies.

Accumulating evidence supports a central role for EBV as a major upstream factor in MS. Longitudinal studies show that EBV infection almost always precedes disease onset and is associated with a marked increase in risk, while altered immune responses to EBV can be detected years before clinical symptoms. However, EBV infection alone is not sufficient to cause MS, and disease development likely reflects the interaction between viral exposure, host susceptibility, and environmental factors. Proposed mechanisms include altered B- and T-cell interactions, persistent immune dysregulation, and molecular mimicry, but these remain incompletely understood and are supported mainly by indirect evidence. In addition, important limitations persist, including the difficulty of detecting EBV in central nervous system compartments and the lack of fully reliable biomarkers. From a prevention perspective, complete avoidance of EBV infection is unlikely to be feasible. A more realistic approach may involve preventing infectious mononucleosis or modifying early immune responses after infection. Progress in this field will require stepwise strategies, combining early-phase studies with long-term evaluation of clinical outcomes.

A key contribution of this review is the integration of epidemiological, immunological, and mechanistic evidence into a clear and practical framework. It distinguishes between strong longitudinal data and more uncertain mechanistic explanations, and connects these insights to prevention strategies such as vaccination, risk stratification, and early intervention. Overall, EBV represents a promising but not yet definitive target for MS prevention. By offering a critical and prevention-focused perspective, this review provides a structured approach to understanding the EBV–MS relationship and supports the development of more realistic and targeted strategies for reducing disease risk.

## Figures and Tables

**Table 1:** Key EBV infection biology checkpoints relevant to neuroimmune disease.

EBV Checkpoint	Mechanism	Neuroimmune Relevance
Primary infection timing and infectious mononucleosis (IM) phenotype	Infection in childhood is often silent, while delayed infection more often causes infectious mononucleosis (IM), with fever, sore throat, swollen lymph nodes, and fatigue; symptoms may persist in some cases [19,20].	Symptomatic infection reflects a stronger inflammatory response; a history of IM is associated with increased MS risk in population studies [17,34,35,36].
Infectious mononucleosis-related immune imprinting	Strong EBV-specific T-cell activation and broad inflammatory signaling may lead to lasting immune changes in some individuals [21,22].	Supports a link between symptomatic infection and long-term immune changes, including low-grade inflammation and fatigue [22].
Latency initiation in B cells	EBV infects naive B cells and induces a growth program that promotes B-cell activation and expansion [14,23,25].	Enables lifelong persistence of EBV within the B-cell compartment [14,23,25].
Germinal center transit	Infected B cells enter germinal centers; EBV mimics survival signals, while cytokines and epigenetic mechanisms regulate viral gene expression [24,25].	Promotes formation of the viral reservoir while reducing immune detection [24,25].
Memory B-cell reservoir	Resting memory B cells express very few viral genes; normal B-cell activation can trigger viral reactivation [23,25].	Supports long-term persistence with minimal antigen exposure, relevant for chronic immune interaction [23,25].
Immune surveillance	Control of EBV mainly depends on CD8 cytotoxic T cells with support from CD4 T cells, especially during primary infection and long-term control [21,37,38,39].	Immune control limits expansion of infected cells and viral reactivation [37,38,39].
Immune evasion	EBV limits viral protein expression and interferes with antigen processing and presentation [13,26].	Reduces detection by T cells and supports viral persistence [13,26].
T-cell dysfunction features	Long-term stimulation may lead to exhaustion-like features, including expression of inhibitory receptors such as PD 1; increased PD1 positive, HLA DR positive CD8 cells are observed in tonsils [27].	May weaken immune control of EBV in certain tissues [27].
Reactivation and human evidence	Reactivation refers to the shift from latency to active viral production; many studies rely on indirect laboratory markers [15].	Serology may remain stable or fluctuate; blood EBV DNA may reflect latent infection rather than active replication; whole blood is often more sensitive than plasma [29,30,31].

**Table 2:** Evidence maps of human study streams linking EBV to MS risk, including primary inferences and key limitations.

Type of Evidence	Key Finding	Primary Limitation
A. Temporal relationship		
Longitudinal studies with repeated samples tracking seroconversion	Temporality and directionality; large risk increase after EBV seroconversion; neuroaxonal injury biomarkers rise after infection [16].	Observational; limited CNS tissue localization; residual confounding and generalizability limits.
Preclinical biobanks (years to decades before onset)	Long preclinical window; EBV immune divergence can predate first symptoms by many years [58,59].	Sampling intervals and assay drift; cannot fully separate trigger from earliest subclinical disease biology.
B. Risk modification (clinical phenotype of primary infection)		
Infectious mononucleosis record linkage cohorts	Risk modification by symptomatic delayed primary infection; delayed risk peak supports a prolonged prodrome [17,35,36].	IM misclassification; detection bias; and confounding from socioeconomic factors and exposure patterns.
Studies of multiple risk factors (IM plus other exposures)	Identifies enriched strata for trial feasibility; supports multi-hit models (EBV plus host and environment) [62,63].	Interaction multiplicity and measurement error; not causal without triangulation or intervention.
C. Immune phenotype and CNS constraints		
High-dimensional serology (peptidome and epitope panels)	Quantitative EBV immune dysregulation beyond EBV-positive vs. EBV-negative; candidate regions for risk enrichment such as EBNA1 [58,59,64].	No single definitive peptide; heterogeneity; cross-reactivity inference often indirect.
CNS humoral studies (paired CSF–serum; intrathecal indices)	EBV-specific antibodies in the CNS are rare, which suggests EBV is more likely a trigger than a persistent driver [65,66].	Indirect measures; low levels do not exclude tissue involvement, and temporary “hit-and-run” effects are still possible.
D. Limits of the hypothesis and supporting evidence		
EBV-seronegative inflammatory demyelination and rare EBV-negative MS	Boundary condition: classic MS is uncommon without EBV exposure; supports a near-necessary factor framing [34,61].	Pediatric spectrum differs from adult MS; assay and timing limits; does not exclude rare EBV-negative pathways.
Genetic causal inference (polygenic scores, Mendelian randomization of EBV traits)	Helps reduce confounding and reverse causation when instruments are valid; strengthens causal inference when aligned with temporality data [62,67,68,69].	Instrument validity and pleiotropy risks; population stratification; not a substitute for interventional proof.

## Data Availability

No new data were created or analyzed in this study. Data sharing is not applicable to this article.
